# A gene network database for the identification of key genes for diagnosis, prognosis, and treatment in sepsis

**DOI:** 10.1038/s41598-023-49311-x

**Published:** 2023-12-09

**Authors:** Qingsheng Li, Lili Qu, Yurui Miao, Qian Li, Jing Zhang, Yongxue Zhao, Rui Cheng

**Affiliations:** Department of Pharmacy, Affiliated Hospital of Inner Mongolia Medical University, Hohhot, Inner Mongolia 010050 People’s Republic of China

**Keywords:** Computational biology and bioinformatics, Immunology

## Abstract

Sepsis and sepsis-related diseases cause a high rate of mortality worldwide. The molecular and cellular mechanisms of sepsis are still unclear. We aim to identify key genes in sepsis and reveal potential disease mechanisms. Six sepsis-related blood transcriptome datasets were collected and analyzed by weighted gene co-expression network analysis (WGCNA). Functional annotation was performed in the gProfiler tool. DSigDB was used for drug signature enrichment analysis. The proportion of immune cells was estimated by the CIBERSORT tool. The relationships between modules, immune cells, and survival were identified by correlation analysis and survival analysis. A total of 37 stable co-expressed gene modules were identified. These modules were associated with the critical biology process in sepsis. Four modules can independently separate patients with long and short survival. Three modules can recurrently separate sepsis and normal patients with high accuracy. Some modules can separate bacterial pneumonia, influenza pneumonia, mixed bacterial and influenza A pneumonia, and non-infective systemic inflammatory response syndrome (SIRS). Drug signature analysis identified drugs associated with sepsis, such as testosterone, phytoestrogens, ibuprofen, urea, dichlorvos, potassium persulfate, and vitamin B_12_. Finally, a gene co-expression network database was constructed (https://liqs.shinyapps.io/sepsis/). The recurrent modules in sepsis may facilitate disease diagnosis, prognosis, and treatment.

## Introduction

Sepsis is a major cause of mortality and morbidity in the intensive care unit^1^. According to the sepsis-3 definition, sepsis is defined as life-threatening organ dysfunction caused by a dysregulated host response to infection^2^. Sepsis can be caused by any type of infection, including bacteria, influenza, pneumonia, and food poisoning^3^. Characterized by systemic inflammatory response syndrome (SIRS) and a suspected or confirmed infection^4^, sepsis can induce fatal medical conditions, such as septic shock accompanied by low blood pressure and a high rate of mortality^4^ and acute respiratory distress syndrome (ARDS)^5^. However, the differences between these sepsis-related diseases are still unclear.

Transcriptome analysis is a cost-effective tool to explore gene expression in sepsis patients, and to develop diagnostic biomarkers and targeted therapies^4,6^. While recombinant human activated protein C (rhAPC) is the sole US Food and Drug Administration (FDA)-approved medicine for severe sepsis treatment^7^, contemporary systems biology methods hold the potential to unravel the underlying disease mechanisms and bridge the gap between research into clinical practice^8^.

Several sepsis studies involve gene-centric transcriptome analysis. For example, gene signatures for severity and endotype were identified from RNA-Seq data^4^. Transcriptome analysis revealed the association of sepsis survival with a robust immune response and the presence of missense variants in VPS9D1^9^. Microarray analysis of sepsis patients with ARDS revealed inflammasome-regulated cytokines such as IL-18 are important in acute lung injury^10^. Upregulated genes related to low-density neutrophils in sepsis leukocytes were identified^11^. Most of these studies used traditional differential gene expression analysis (DGE) that predominantly focused on individual genes, leading to challenges in result reproducibility due to gene functional redundancy across studies. In contrast, module-based analysis, which centers on clusters of co-expressed genes, has proven to be more reliable than single gene analysis^12^. Therefore, we collected six independent datasets and used the module-based analysis to find recurrent modules.

Here, we applied weighted gene co-expression network analysis (WGCNA) on the largest RNA-Seq sepsis dataset GSE185263 to identify reference modules, to which other datasets can be projected. We found four critical modules associated with cell cycle, neutrophil activation, and natural killer cell mediated cytotoxicity. These modules effectively distinguish between patients with varying survival times. Furthermore, our analysis unveiled sepsis-related drugs, some of which are well-supported by existing literature. Our analysis offers significant insights into sepsis diagnosis, prognosis, and treatment.

## Results

### Co-expressed gene modules identified in sepsis

**Figure 1:**
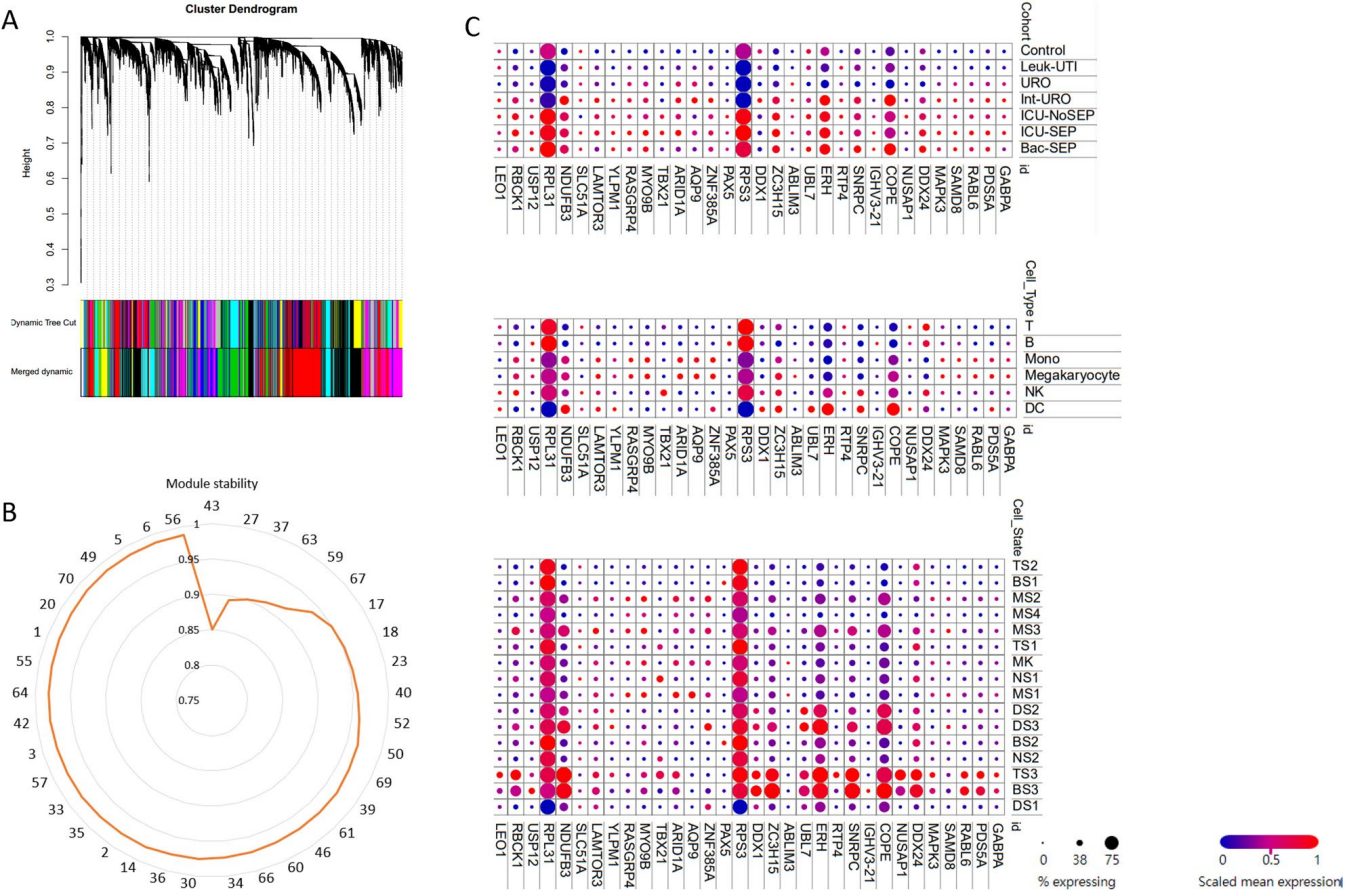
Gene modules identified in the sepsis RNA-Seq dataset GSE185263 are stable. (**A**) Cluster dendrogram showing the cluster tree and module assignment. Each module is denoted with different colors. (**B**) Radar plot showing the correlation of the original module connectivity and the sampled one for each module. (**C**) Expression of hub genes in each module in different diseases, cell types, and cell status. *TS* T cell subclusters, *NS* NK cell subclusters, *MS* monocyte subclusters, *MK* megakaryocyte subclusters, *DS* dendritic cell (DC) subclusters, *BS* B cell subclusters.

Although sepsis transcriptome has been profiled in several studies, the small sample size and microarray platform limit the analysis power. We used the currently largest sepsis RNA-Seq dataset GSE185263 to construct a gene co-expression network (Fig. 1A). Using the WGCNA method, a total of 37 co-expressed gene modules were identified (Table 1). To test if these modules are stable, the module stability analysis was performed. Results showed that the average module stability was larger than 0.96 with a standard deviation of 0.015 (Fig. 1B). Functional enrichment analysis revealed that these modules were associated with transcription factors, translation, immune response, cell cycle, mitochondrion, leukocyte activation, platelet activation, and interferon signaling (Table 1). These modules are associated with SARS-CoV-2 infection, except that M42 is associated with mouse hepatitis virus (MHV) infection, M50 and M57 are associated with SARS-COV-2 infected mouse heart, and M70 is associated with SARS-CoV-2 and seasonal coronavirus host factors. While M60 is novel, it is not enriched in any diseases. Figure 1C shows the differential expression patterns of module hub genes across disease status, cell types, and cell status. All the module genes and related information is provided in Supplementary Table 1.

**Table 1:** Functional and immune cell annotation of the identified modules.

Module (size)	Functional annotation	Hub gene	Immune cell
1 (1784)	Factor: HDAC2 (1E−56) hsa-miR-21-5p (1E−35) nucleus (1E−29)	GABPA	Th1 CD4^+^ T cell (–)
2 (2572)	Factor: HDAC2 (7E−41) Nucleic acid metabolism (1E−37) nucleus (2E−33)	PDS5A	CD4^+^ T cell
3 (1592)	Factor: SP2 (6.4E−58) Factor: ZF5 (1E−57)	RABL6	Hematopoietic stem cell (–)
5 (1438)	Factor: ETF (5E−18) hsa-miR-21-5p (3E−10) whole membrane (3E−9)	SAMD8	Neutrophil; Th1 CD4^+^ T cell (–)
6 (919)	Factor: Sp1 (1E−34) Factor: ETF (1E−32)	MAPK3	NKT; Memory CD4^+^ T cell (–)
14 (993)	Generation of second messenger molecules (1E−20) Adaptive immune response (2E−16)	DDX24	CD8^+^ T cell
17 (248)	Cell cycle (1E−87) hsa-miR-193b-3p (3E−37) Kinetochore (1E−36)	NUSAP1	Th2 CD4^+^ T cell; Neutrophil (–)
18 (245)	Factor: Elf-1 (0.006)	RNU7-181P	
20 (1160)	Factor: ER81 (7E−38) Factor: ZF5 (1E−36)	COPE	Hematopoietic stem cell (–)
23 (377)	Erythrocyte differentiation (7E−7)	TRIM10	
27 (160)	Antigen binding (1E−171) Adaptive immune response (6E−121)	IGHV3-21	Plasma B cell
30 (290)	Mitochondrion (5E−28) Mitochondrial translation (2E−20)	SNRPC	Th1 CD4^+^ T cell; Neutrophil (–)
33 (1139)	Myeloid leukocyte activation (2E−18)	C3orf86	Neutrophils; CD8^+^ T cell (–)
34 (225)	Defense response to virus (9E−40) Interferon Signaling (7E−34) Innate immune response (5E−33)	RTP4	Neutrophil
35 (151)	Mitochondrial inner membrane (1E−26) Factor: ER71 (2E−15)	ERH	Common lymphoid progenitor
36 (112)	Factor: SP1 (1E−7)	UBL7	
37 (166)	Platelet activation, signaling and aggregation (6E−18)	ABLIM3	
39 (101)	Factor: Erg (2E−6) Organelle lumen (9E−6)	ZC3H15	Common lymphoid progenitor
40 (169)	ncRNA metabolism (5E−15)	DDX1	Common lymphoid progenitor; Neutrophil (–)
42 (92)	Formation of a pool of free 40S subunits (2E−91) Protein targeting to ER (4E−86) Viral mRNA Translation (4E−84)	RPS3	Neutrophil (–)
43 (89)	B cell receptor signaling pathway (1E−5)	PAX5	B cell; Monocyte (–)
46 (82)	Plasma membrane (0.002)	ZNF385A	
49 (65)	Exocytosis (2E−19) Neutrophil activation involved in immune response (5E−19)	AQP9	Neutrophil; B cell (–)
50 (60)	Chromatin organization (3E−7) hsa-miR-218-5p (4E−6)	ARID1A	
52 (58)	Natural killer cell mediated cytotoxicity (3E−6) Factor: MAFA (0.02)	TBX21	
55 (58)	hsa-miR-484 (3E−5) Factor: CTC (8E−5)	MYO9B	Common lymphoid progenitor
56 (316)	Neutrophil activation (2E−12)	RASGRP4	Neutrophil; Central memory CD8^+^ T cell (–)
57 (51)	Chromatin organization (5E−12)	YLPM1	CD4^+^ T cell
59 (49)	Neutrophil degranulation (3E−23); Neutrophil activation (5E−22)	CEACAM6	Common myeloid progenitor; CD8^+^ T cell (–)
60 (47)			
61 (87)	Factor: ER81 (7E−6)	LAMTOR3	
63 (45)		SLC51A	Endothelial; CD4^+^ T cell (–)
64 (45)	Peptide disulfide oxidoreductase activity (0.006)	NDUFB3	CD4^+^ T cell (–)
66 (38)	Eukaryotic translation elongation (1E−37) Selenocysteine synthesis (7E−35) Influenza Infection (4E−30)	RPL31	
67 (36)	hsa-miR-218-2-3p (0.01)	USP12	
69 (34)	Interferon alpha/beta signaling (6E−7)	RBCK1	pDCs
70 (31)	Ribosome biogenesis in eukaryotes (0.01)	LEO1	Neutrophil (–)

*pDCs* plasmacytoid dendritic cells (pDCs); (–): the immune cell proportion is negatively associated with module expression with an absolute R > 0.6 and P < 0.01.

### Modules were associated with immune cell populations

To check if these identified modules were associated with immune cell proportion, CIBERSORT was used to deconvolute GSE185263 into immune cell proportion. Correlation analysis between immune cell proportion and module expression indicates the links between them (top assignment with an absolute R > 0.6 and P < 0.01, Table 1). These results were confirmed in a single-cell transcriptome dataset. For example, IGHV3-21 is the hub gene of M27 and is highly expressed on the BS3 sub-cluster which is composed of B cells (Fig. 1C). Indeed M27 expression was significantly and positively correlated with plasma B cell abundance. In several modules (M5, M17, M30, M33, M49, and M56), the reverse correlation between neutrophils and, B cell, T cells was observed. This may indicate that neutrophils suppress T cell proliferation.

### Modules were differentially expressed in sepsis and control

**Figure 2:**
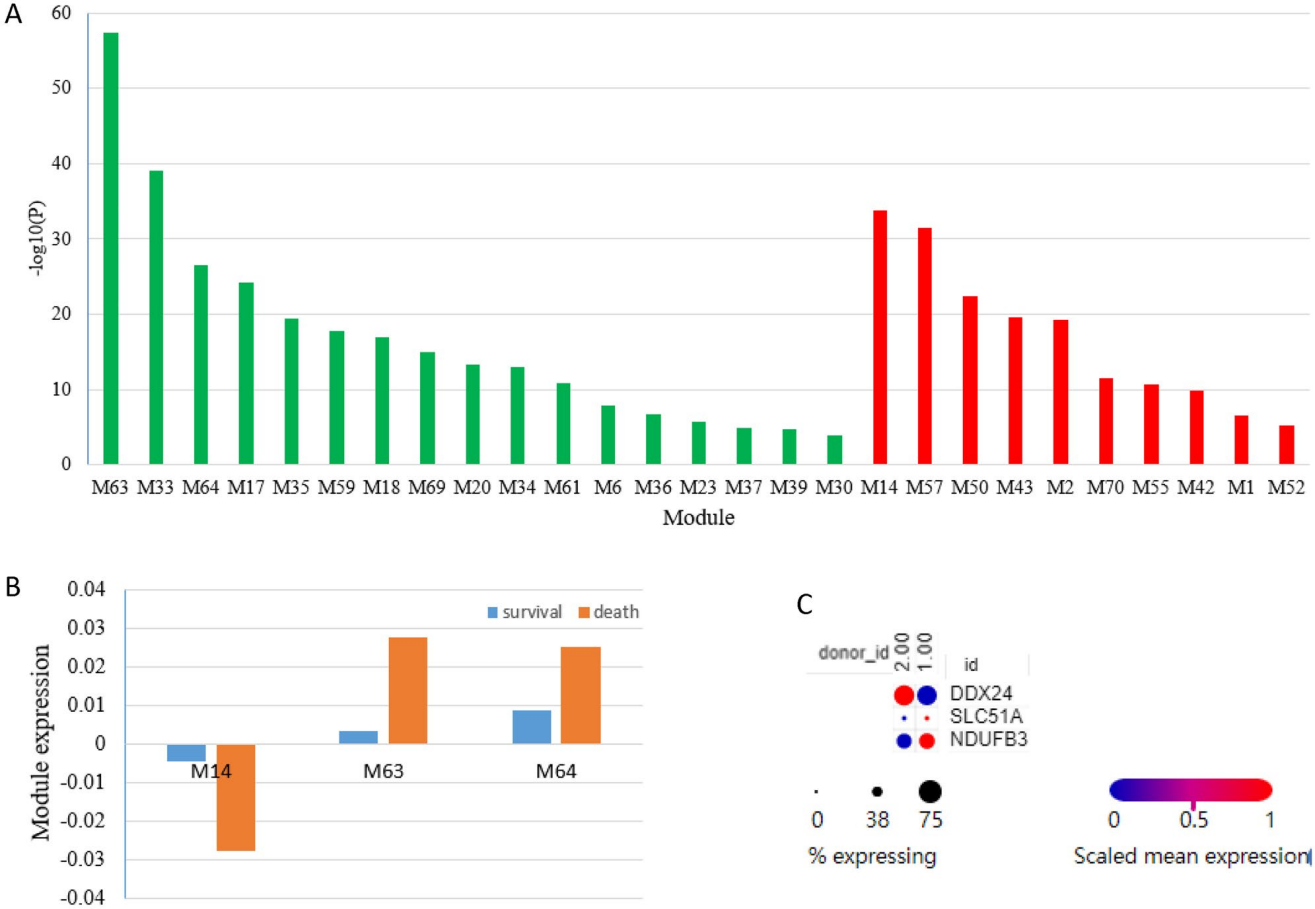
Significantly changed modules in sepsis RNA-Seq dataset GSE185263. (**A**) The sepsis transcriptome was compared with normal control. Green bars indicate upregulated modules. Red bars indicate downregulated modules. The statistical significance was set at 0.01/38. (**B**) non-survived patients were compared with survived patients. (**C**) The expression of hub genes of M14, M63, and M64 was confirmed in another sepsis dataset.

**Figure 3:**
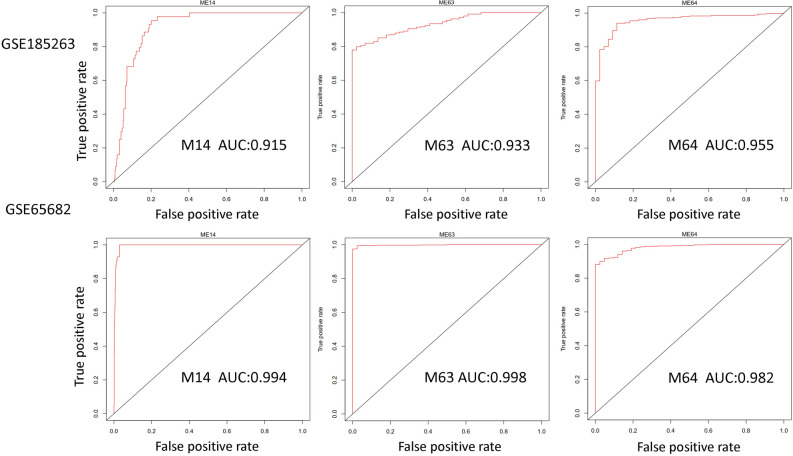
Modules M14, M63, and M64 can recurrently discriminate samples of sepsis and control in both datasets GSE185263 and GSE65682.

As the high dimensional sepsis transcriptome data has been reduced to tens of modules, module expression was compared between different sepsis groups. We found that 27 modules were differentially expressed between sepsis and normal groups. Among them, 17 modules were up-regulated and 10 modules were down-regulated in sepsis compared to control (Fig. 2A). Modules M14, M63, and M64 were also significantly changed in non-survived patients compared with survived patients (Fig. 2B). The expression patterns of hub genes of these modules were confirmed in another dataset (Fig. 2C). The three modules could perfectly discriminate samples of sepsis and control in both datasets GSE185263 and GSE65682 (Fig. 3). Other modules including M2, M33, M35, M57, and M63 had an AUC larger than 0.9.

### Modules were differentially expressed in SIRS, sepsis, septic shock, and ARDS

**Figure 4:**
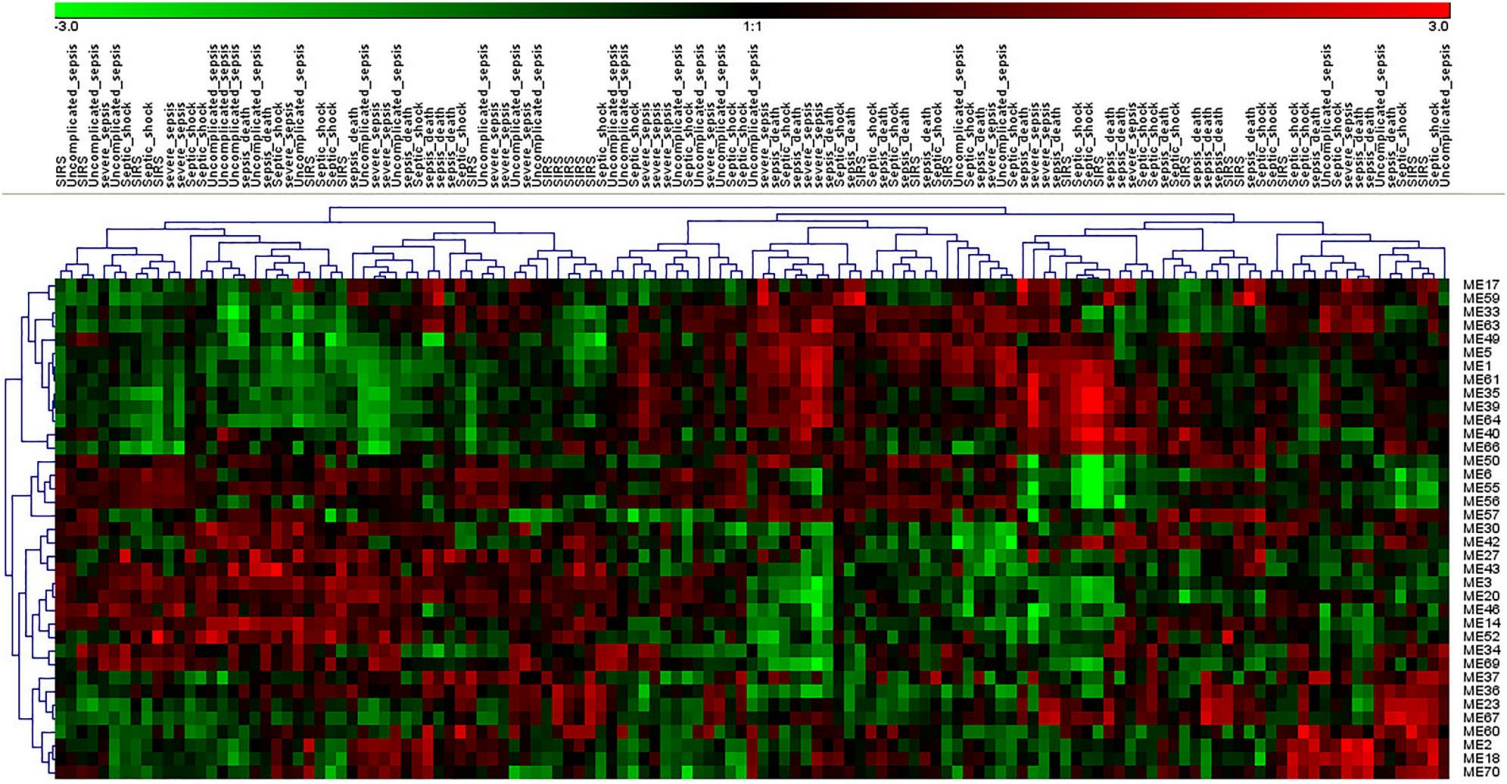
Modules had different expression patterns in SIRS, sepsis, and septic shock.

Severe sepsis or septic shock is characterized by an excessive inflammatory response to infectious pathogens^5^. We analyzed the dataset GSE63042 which contains transcriptome data for SIRS, sepsis, septic shock, and sepsis death. Multivariate analysis of variance (MANOVA) was applied to dataset GSE63042. M33 was upregulated in severe sepsis, sepsis shock, and sepsis death compared to SIRS. M52 was downregulated in severe sepsis and sepsis death compared to SIRS. M57 was upregulated in sepsis death compared to severe sepsis. M59 was upregulated in sepsis death compared to SIRS, uncomplicated sepsis, and septic shock. M63 was upregulated in severe sepsis, septic shock, and sepsis death compared to SIRS (Fig. 4).

ARDS is a devastating complication of severe sepsis, which results in a high mortality rate. We analyzed the dataset GSE32707 and found that M27, M49, M59, and M63 were upregulated and M30, M35, M39, M40, M42, M60, M66, and M70 were downregulated in day 0 ARDS compared to normal control.

### Modules were associated with the survival of critically ill patients with sepsis

**Figure 5:**
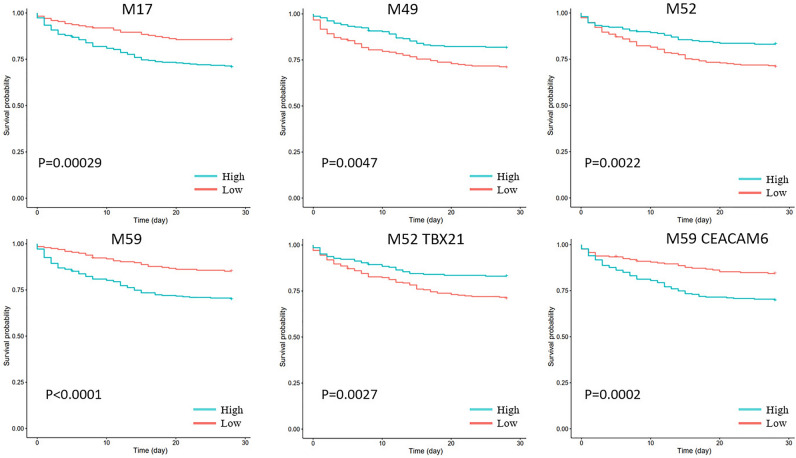
Modules M17, M49, M52, and M59, and hub genes TBX21 and CEACAM6 were associated with patient survival after adjusting for endotype class and age. The analysis was performed in dataset GSE65682.

To check if the identified modules are associated with survival, we performed survival analysis in the dataset GSE65682 of critically ill patients with sepsis. Modules M17, M49, M52, and M59 were found to be associated with patient survival after adjusting for endotype class and age (Fig. 5). Hub genes of these modules were also associated with patient survival (Fig. 5). In sepsis dataset GSE185263, we found that M52 (R = − 0.34, P = 8E−11) and M60 (R = 0.35, P = 7E−12) had the highest absolute correlation value with Sequential Organ Failure Analysis (SOFA) score. M60 is enriched with non-coding genes. Survival analysis for all the modules can be explored at the sepsis gene co-expression database (https://liqs.shinyapps.io/sepsis/).

### Module expression was changed in severe A (H1N1) pandemic influenza

**Figure 6:**
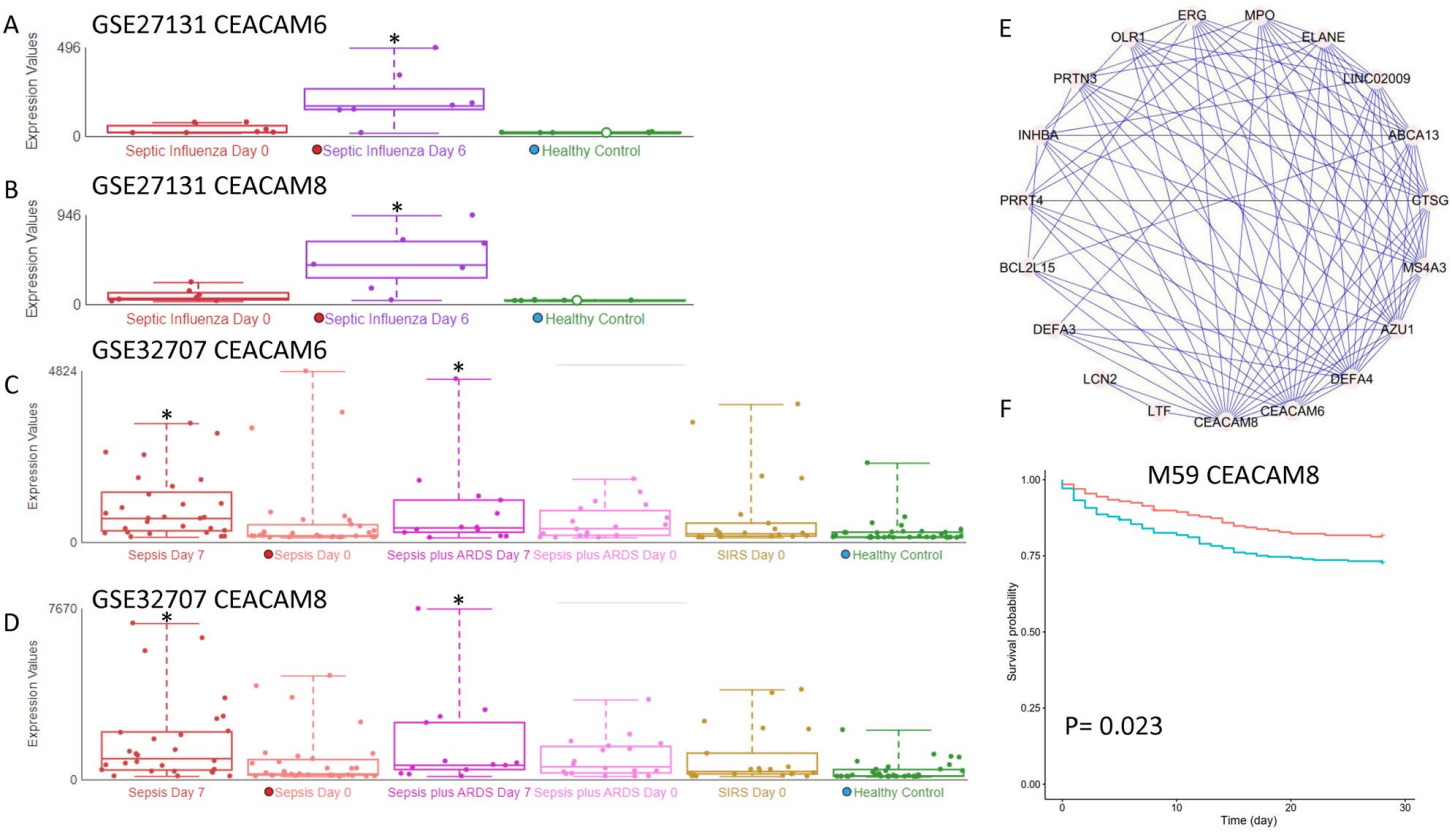
M59 hub gene CEACAM6 was highly co-expressed with CEACAM8, which is also prognostic for survival. (**A**) CEACAM6 was up-regulated in day 6 septic influenza patients. (**B**) CEACAM8 was up-regulated in day 6 septic influenza patients. (**C**) CEACAM6 was up-regulated in day 7 septic ARDS patients. (**D**) CEACAM8 was up-regulated in day 7 septic ARDS patients. (**E**) The top 100 connections in M59. (**F**) CEACAM8 can separate patients with long and short survival.

Critically ill patients with sepsis caused directly by influenza viruses, or by influenza-induced secondary bacterial infections are increasing worldwide. Therefore, we performed differential analysis for GSE27131 to examine if these modules are associated with disease development in severe A (H1N1) pandemic influenza. ME59 was found to be strongly up-regulated on day 6 compared to day 0 (P = 0.003), but not changed on day 0 compared to the control. Thus, M59 was associated with severe influenza development. We found that the M59 hub gene CEACAM6 and its co-expressing CEACAM8 were both up-regulated in the influenza dataset (Fig. 6A,B). CEACAM6 has been reported as a protein receptor for the influenza A virus^13^. Interestingly, CEACAM6-high airway neutrophils and epithelial cells are a feature of severe asthma^14^. In our analysis, the module was associated with neutrophil activation (P = 5E−22), indicating CEACAM6 as a potential biomarker for severe influenza that requires mechanical ventilator support. The finding was confirmed in another dataset GSE32707, in which both day 0 and day 7 sepsis patients with acute respiratory distress syndrome (ARDS) had stronger M59 expression compared to control (P < 0.0001)^10^. Genes CEACAM6 and CEACAM8 were also confirmed to be up-regulated (Fig. 6C,D). In M59, many genes were co-expressed with CEACAM6 (Fig. 6E), such as CEACAM8 which can also separate patients with long and short survival (Fig. 6F).

### Modules can separate patients with severe community-acquired pneumonia

**Figure 7:**
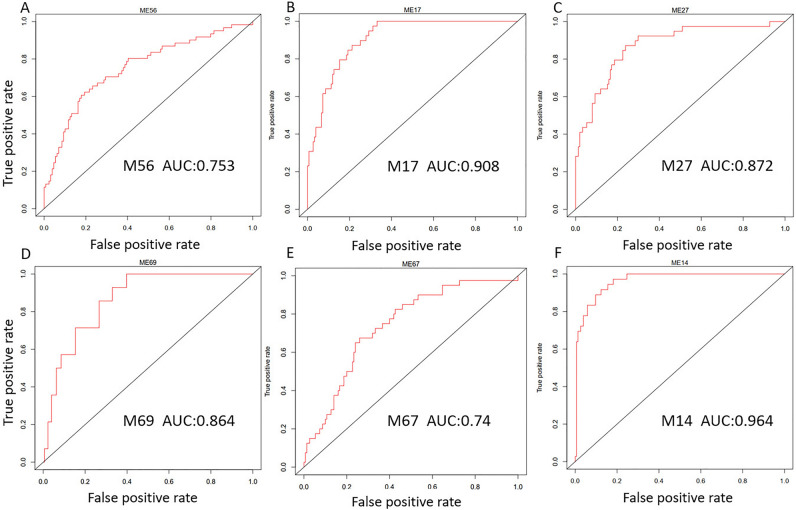
Modules can separate pneumonia patients with different etiologies. The analysis was performed in GSE40012. (**A**) Bacterial pneumonia. (**B**,**C**) Influenza pneumonia. (**D**) Mixed bacterial and influenza A pneumonia. (**E**) SIRS. (**F**) Health control.

Diagnosis of severe pneumonia remains challenging because of a lack of correlation between the clinical status and etiology^15^. Causes of severe respiratory failure include bacterial pneumonia, influenza pneumonia, mixed bacterial and influenza A pneumonia, and non-infective SIRS. We used dataset GSE40012 to test if the modules can separate pneumonia patients with different etiologies. For each disease, we selected the top one or two modules to describe here. For example, M56 can separate bacterial pneumonia from other pneumonia (Fig. 7A). M17, M27, and M59 can separate influenza pneumonia from other pneumonia (Fig. 7B,C). M69 can separate mixed-type pneumonia from other pneumonia (Fig. 7D). M67 can separate SIRS from other pneumonia (Fig. 7E). M14 can separate health control from pneumonia (Fig. 7F).

### Candidate drugs associated with sepsis

**Figure 8:**
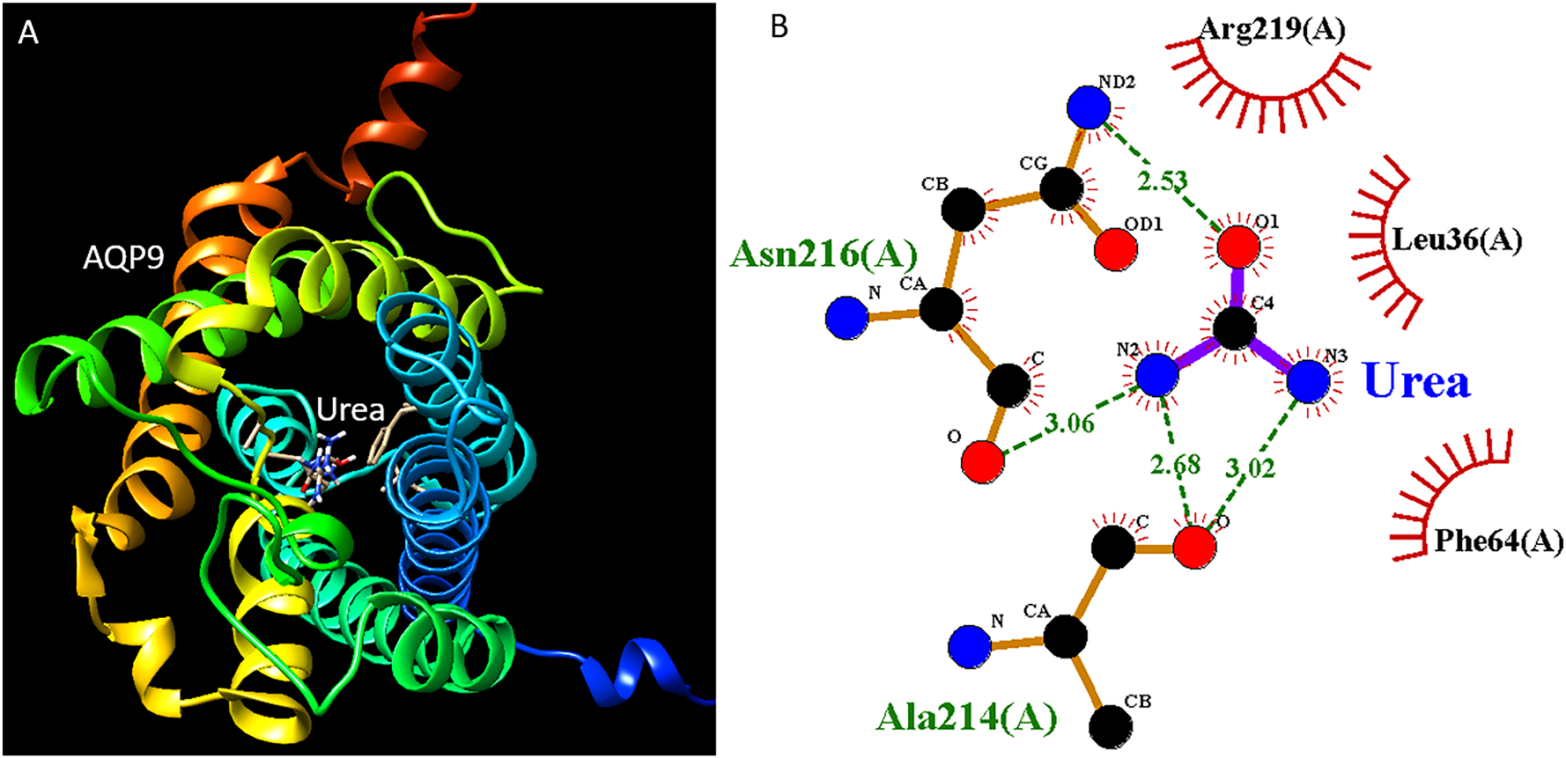
Docking analysis for M49 hub gene AQP9 in complex with urea. (**A**) AQP9 in complex with urea. (**B**) The pose view shows the interaction sites.

Four modules M17, M49, M52, and M59 were associated with patient survival. We searched the genes of these modules in DSigDB to identify related chemicals. We found that M17 was enriched with testosterone (P = 3E−145) and phytoestrogens (P = 2E−55) signature genes. M49 was enriched with Ibuprofen (P = 6E−4) and urea (P = 8E−4) signatures genes. M52 was enriched with Dichlorvos (P = 1E−6) and ZIRAM (P = 1E−6) signatures genes. M59 was enriched with Potassium persulfate (P = 2E−5) and adenylyl sulfate (P = 4E−4) signatures genes. GWAS Catalog analysis revealed that M49 and M59 genes were associated with monocyte percentage of white cells (P = 0.004) and vitamin B_12_ levels (P = 0.007) respectively. We also performed docking analysis to the hub gene of M49 AQP9 in complex with urea (Fig. 8A). The interaction may occur at sites Ala214 and Asn216 (Fig. 8B).

### A user-friendly database for sepsis gene co-expression was constructed

**Figure 9:**
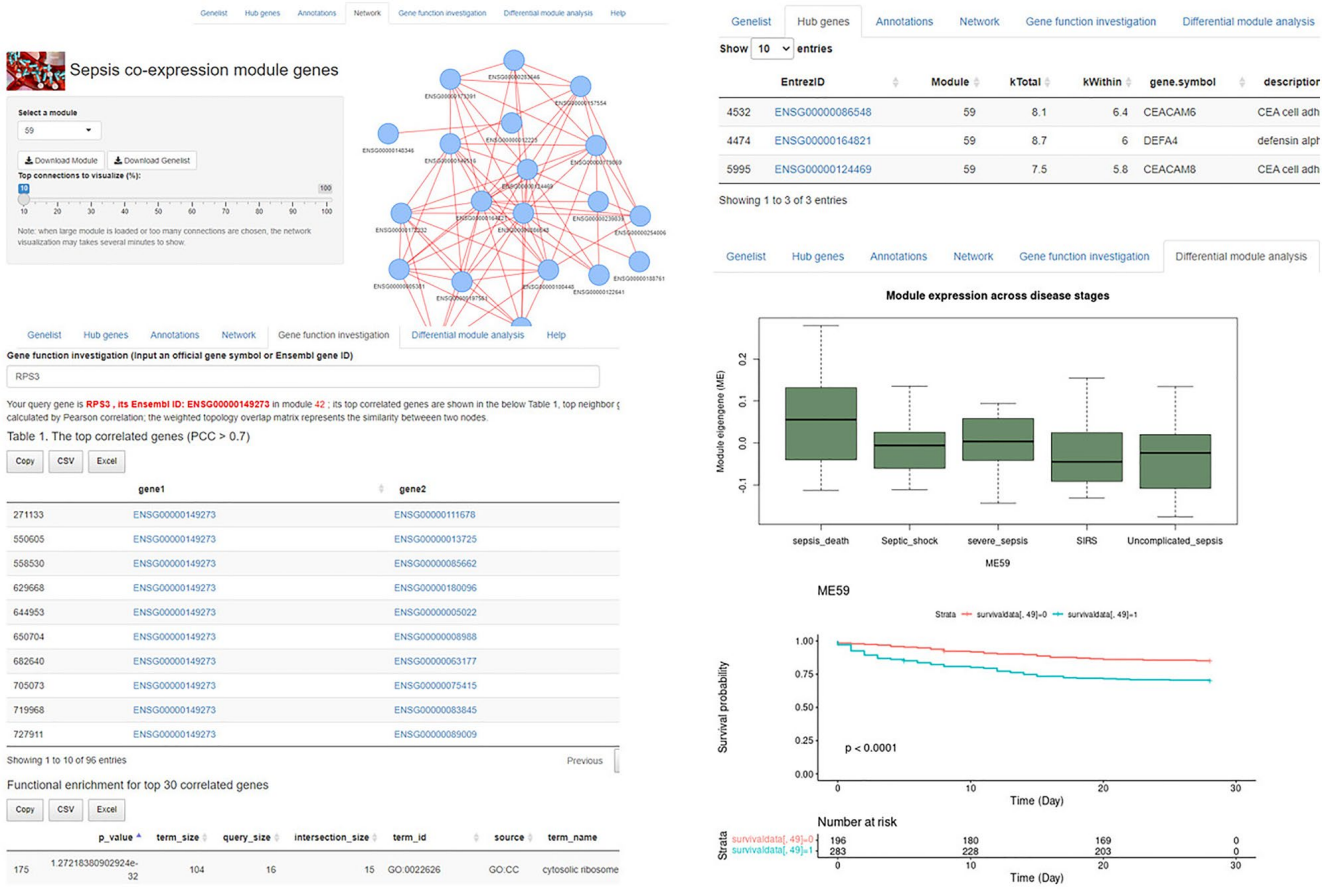
An easy-to-use gene co-expression database for sepsis was constructed.

Although sepsis transcriptomes have been analyzed in several publications, there are still few web tools available for researchers. Thus, we for the first time constructed an easy-to-use web tool that provides informative data about the above sepsis gene co-expression network. The modules of the database include module gene list, module hub gene list, module network visualization, gene function investigation, and differential module analysis (Fig. 9). The database is available at https://liqs.shinyapps.io/sepsis/. The database will provide a useful tool for researchers to generate testable hypotheses.

## Discussion

We first applied WGCNA to the currently largest RNA-Seq sepsis dataset and identified 37 modules. The clinical relevance of these modules was validated in sepsis, sepsis shock, SIRS, ARDS, and severe influenza A patients. Although gene co-expression networks have been constructed in several studies, they are limited to sepsis samples from the microarray platform^1,16^. For example, two studies used the same method and dataset but got results with few overlapped genes^1,17^. It has been reported that module-based analysis will give more consistent results^18^. Therefore, we comprehensively analyzed the sepsis network and provided detailed information about the modules.

We used the RNA-Seq dataset GSE185263 for reference module identification and projected transcriptome data from the other 5 datasets to them. Based on these modules, we can reduce the high dimensional transcriptome data to tens of modules, and then correlate modules with clinical data to infer important modules. Among the identified modules, two modules M34 and M69 were related to innate immunity, and two modules M30 and M35 were related to mitochondrion. These modules were annotated with immune cells. With this information, the modules, immune cell types, and clinical parameters can be linked. For example, M30 was annotated as Th1 CD4^+^ T cell and was upregulated in ARDS. Increased mitochondrial biogenesis can prolong CD4^+^ T cell activation, indicating the critical role of M30 in ARDS^19^. M33 was enriched with activated myeloid leukocytes and was annotated as neutrophils and CD8^+^ T cells (–). Neutrophils can suppress CD8^+^ T cells^20^. M33 was upregulated in sepsis, whose expression was negatively correlated with CD8^+^ T cell proportion, indicating the role of M33 in the dysfunctional/decreased CD8^+^ T cells. Interestingly, PD-1 expression was also upregulated in sepsis (data not shown), indicating the unfavorable immune status with decreased and exhausted CD8^+^ T cells in sepsis^21^. M43 was enriched with the B cell receptor signaling pathway and was annotated as B cell and monocyte (–). M43 was downregulated in sepsis, indicating the increased monocyte in sepsis. Inflammatory monocytes can hinder antiviral B cell responses^22^. M63, the most significantly upregulated module in sepsis, was annotated as endothelial and CD4^+^ T cell (–). Endothelial are activated in sepsis^23^. It has been reported that activated murine lung endothelial can induce Tregs to suppress CD4^+^ T cell proliferation^24^. M69, upregulated in sepsis, was annotated as pDCs and involved in Interferon alpha/beta signaling. pDCs are a specialized cell type producing natural interferon (IFN)^25^. Increased circulating pDCs during sepsis but decreased pDCs in nonsurvivors compared to survivors was observed, indicating it may function as an early predictive biomarker for the outcome of sepsis^26^.

Hub genes of the modules are important in sepsis with literature support. For example, the Hub gene of M43 PAX5 is the guardian of B cell identity and function^27^. TBX21 expression in M52 is inversely associated with SOFA and mortality^28^. SLC51A in M63 is identified as a reduced mortality signature^4^. AQP9 in M49 can regulate neutrophil cell migration and impact sepsis survival^29^. Mutation of RBCK1 in M69 is associated with recurrent episodes of sepsis in children and causes death^30^. E3 ubiquitin ligase gene TRIM31 is involved in the development of sepsis^31^. TRIM10 in M23 is of the same gene family member, but with unexplored roles in sepsis.

Many biomarkers based on a single gene cannot be reproduced across studies, even studies with the same datasets analyzed. We used a more stable module to discriminate samples. The modules could separate sepsis and control samples reproducibly in two independent datasets GSE185263 and GSE65682. In the pneumonia dataset GSE40012, M17 had the highest ability to separate influenza pneumonia from other pneumonia. M17 is involved in the cell cycle. A previous study showed that cell-cycle regulation genes were signatures for influenza, which is different from that caused by bacterial pathogens or SIRS^15^. This information suggests that our results are reliable. However, due to the small sample size, the result of modules separating SIRS, sepsis, sepsis shock, and ARDS is not satisfactory. Current literature also does not provide a satisfactory molecule for discrimination of these diseases, which may be attributed to the inherent heterogeneity of the complex disease^6^. We used the module-centric method to overcome the problem as modules are more stable than genes. Besides, a cohort with a larger sample size should be used when aiming to address such issues.

In the candidate drugs analysis, we found that many of the results have related literature support. For example, M17 was associated with testosterone and phytoestrogens. Testosterone is the major sex hormone in males, which plays a key role in immune depression^32^. It has been reported that the hospital mortality rate was higher in male than in female patients^32^. In mice, the depletion of testosterone can improve survival after polymicrobial sepsis^32^. Interestingly, in dataset GSE65682, high M17 was associated with short survival of sepsis patients. Therefore, testosterone can be a useful marker and target for disease prognosis and treatment. Isoflavones are a common type of phytoestrogens. Soy isoflavone daidzein pretreatment can improve survival in a mouse model of sepsis^33^. Hub gene of M17 NUSAP1 can be downregulated by 17b-estradiol and soymilk which contains a large quantity of daidzein^34^, indicating the potential mechanism of action. High M49 expression patients have longer survival. M49 was associated with ibuprofen and urea. Ibuprofen has been applied in treating patients with severe sepsis^35^, however, its mechanism is still unknown^36^. Daily ibuprofen increased the kidney urine output by increasing some phosphorylated forms of AQP2^37^. The hub gene of M49 is AQP9 which is involved in urea elimination^38^. Thus, urea may play a negative role in sepsis and can be a potential biomarker. Interestingly, a recent report concluded that blood urea nitrogen (BUN) level is independently and positively linked with the presence and severity of sepsis in neonatal^39^. Thus, urea may play a key role in sepsis development, and may serve as a treatment target. M52 was associated with dichlorvos and ZIRAM (fungicide). Dichlorvos is a highly hazardous pesticide that can cause sepsis^40^. M59 was associated with potassium persulfate and adenylyl sulfate. Critically ill patients with abnormal K^+^ levels had a higher incidence of ICU mortality than patients with normal K^+^ levels^41^. Besides, M49 and M59 were also associated with monocyte percentage of white cells and vitamin B_12_ levels. Interestingly, monocyte counts were independently associated with mortality in patients with sepsis^42^. Serum vitamin B_12_ levels have been positively correlated with increased mortality in critically ill patients^43^. Thus, our analysis generated novel hypotheses that merit future validation. These drugs may provide new treatment options for sepsis.

## Conclusion

In conclusion, our study identified prognostic modules, hub genes, and drugs for sepsis. These modules had good discrimination ability in multiple datasets. A sepsis gene co-expression database was first developed. Our analysis provides important information for the sepsis study.

## Material and methods

### Sepsis datasets

Six sepsis datasets were retrieved from the National Center for Biotechnology Information (NCBI) Gene Expression Omnibus (GEO) database^44^. Two of the datasets were from RNA-Seq and four were from gene expression microarray. Dataset GSE185263 currently is the largest RNA-Seq sepsis dataset, with 348 sepsis samples and 44 healthy controls^4^. Detailed information about these datasets is shown in Table 2. Expression matrices for these datasets were downloaded directly from the Series Matrix File(s) link provided in the GEO database. The GSE185263 expression matrix was filtered with a mean count > 10 and a standard deviation > 0.1 before downstream analysis. Finally, 15,688 genes were retained for gene co-expression network analysis. For other datasets, all the gene IDs were converted to Ensembl as the ID types across datasets were different.

**Table 2:** Information for the datasets analyzed in the study.

GEO ID	Patient cohort	Transcriptome platform
GSE185263	348 sepsis, 44 healthy	Illumina HiSeq 2500
GSE63042	106 sepsis, 23 SIRS	Illumina Genome Analyzer II
GSE32707	58 sepsis, 31 ARDS, 21 SIRS, 34 healthy	Illumina HumanHT-12 V4.0 expression beadchip
GSE65682	760 critically ill patients with sepsis, 42 healthy	Affymetrix Human Genome U219 Array
GSE27131	7 with severe A (H1N1) pandemic influenza, 7 healthy	Affymetrix Human Gene 1.0 ST Array
GSE40012	114 with severe community-acquired pneumonia, 40 SIRS, 36 healthy	Illumina HumanHT-12 V3.0 expression beadchip

### Gene co-expression module identification

Weighted gene co-expression network analysis (WGCNA) was performed to identify gene co-expression modules. According to the WGCNA R package manual^45^, parameters were set as following: softPower = 16, corOptions =  “use = '‘p’”, networkType =  “signed”, minModuleSize = 30, deepSplit = 4, MEDissThres = 0.2. It has been reported that rank-based networks require a lower power parameter to achieve a scale-free network^46^. Another advantage of this conversion is that novel transcriptomes can be projected to the reference modules to calculate module-level expression values^12^. Therefore, we converted the expression values into ranks before network analysis. Pearson correlation coefficient was calculated for each gene in the gene rank matrix. An adjacency matrix was constructed by raising the correlation matrix to a power of 16. This step generated a scale-free network, which is a common property of the biology network. The weighted network was transformed into a network of topological overlap (TO)—a metric that measures not only the correlation of two genes but also the extent of their shared correlations across the weighted network. Genes were hierarchically clustered based on their TO. Finally, co-expression gene modules were identified by the Dynamic Tree Cut algorithm^45^. As genes in a module are highly correlated, module genes can be summarized as a module eigengene (ME) by singular value decomposition^47^. WGCNA provides information about gene module assignment, gene connectivity, and module expression information. Connectivity is the sum of correlations of a gene with all other genes in the module or network. Hub gene in a co-expression module tends to have the highest connectivity. Module stability was tested for each module by half sampling 1000 times and was represented by a correlation of intra-module connectivity between the original one and the sampled one in the form of mean ± standard deviation^48^. The module-level expression for other datasets was calculated by the moduleEigengenes function.

### Module annotation

Functional enrichment analysis of modules was performed in the gProfileR package^49^. For simplicity, only representative terms with significance P < 0.01 were recorded. CIBERSORT tool was used to estimate the immune cell proportions in the blood from transcriptome data^50,51^. ME matrix was correlated with the immune cell proportion matrix by Spearman correlation. For each module, only the top correlated immune cell type with an absolute correlation R > 0.6 was kept. Drug Signatures Database (DSigDB) was used to convert module gene sets into drugs^52^. Enrichr was used to check the overlap of the module genes with known disease gene sets^53^. GWAS Catalog was used to test if the known SNPs in a module gene set were associated with phenotypes^54^. The significance was set at 0.05.

### Statistical analysis

The Student’s *t*-test was performed when comparing two groups of expression values. The significance value was adjusted as 0.05/n, where n indicates the times comparison performed according to the Bonferroni correction. Analysis of variance (ANOVA) was performed when comparing expression data from more than two groups for a single dependent variable. Multivariate Analysis of Variance (MANOVA) was performed when comparing expression data from more than two groups for multiple dependent variables. An adjusted P value smaller than 0.05 was considered statistically significant. The Student’s *t*-test ANOVA, and MANOVA were performed in R package base, car, and stats^55^.

### Supplementary Information

Supplementary Table 1.

## Data Availability

The data in the study are available in the Gene Expression Omnibus (GEO) (https://www.ncbi.nlm.nih.gov/geo/).
